# Determination of molecular hydration in solution *via* changes in magnetic anisotropy†

**DOI:** 10.1039/d3cc00601h

**Published:** 2023-03-20

**Authors:** Marcus J. Giansiracusa, Michele Vonci, Yasmin L. Whyatt, Carys Williams, Kevin Mason, David Parker, Eric J. L. McInnes, Nicholas F. Chilton

**Affiliations:** a Department of Chemistry, The University of Manchester Manchester M13 9PL UK eric.mcinnes@manchester.ac.uk nicholas.chilton@manchester.ac.uk; b Department of Chemistry, Durham University Durham DH1 3LE UK; c Department of Chemistry, Hong Kong Baptist University Kowloon Tong Hong Kong davidparker@hkbu.edu.hk

## Abstract

The hydration behaviour of coordination complexes is important for understanding their roles as bio-imaging agents. Determination of hydration is difficult, and various optical and NMR-based techniques have been used. Here we use EPR spectroscopy to unambiguously demonstrate that a *t*-butyl-pyridyl-functionalised Er^III^ DOTA derivative coordinates water, while its methylphosphinate analogue does not.

## Abstract 2

The hydration behaviour of coordination complexes is important for understanding their roles as bio-imaging agents. We use EPR to determine that a carboxylate PARASHIFT reagent coordinates water while the phosphinate analogue does not.

## Graphical abstract

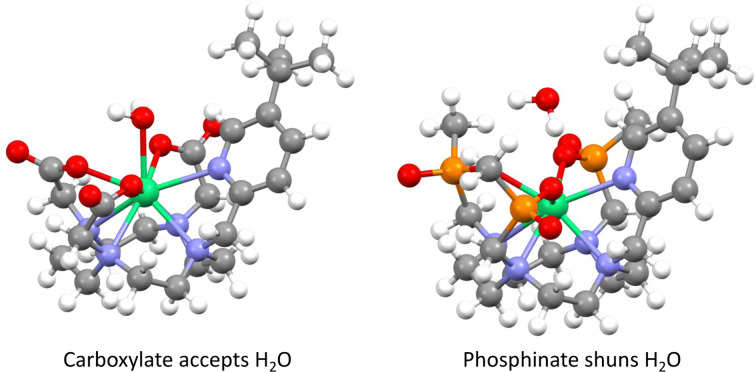

Hydration of lanthanide complexes *in vivo* is a crucial consideration for the performance of inorganic biomedical imaging agents, whether optical^1–3^ or in magnetic resonance imaging (MRI),^4^ and thus determination of hydration behaviour in aqueous media is key when developing new probes. This is important, not only due to the changes in nuclear relaxation times associated with metal coordination in MRI,^5–7^ but also due to the profound influence hydration can have on magnetic anisotropy, and hence on spectral features.^6,8,9^ A common approach to this task involves luminescence lifetime determination, where the optical lifetime of the metal-localised excited state is determined in water, and then remeasured in deuterated water: the relative quenching effect of the O–D *vs.* O–H stretching modes then allows an approximation of the hydration number to be made.^10,11^ However, this method is only pertinent for luminescent complexes and gives no indication of the structure of the complex. Another method to determine hydration involves nuclear magnetic resonance (NMR) spectroscopy, where a change in symmetry alters the diamagnetic spectrum, whereas a change in magnetic anisotropy for paramagnetic complexes can drastically alter the pseudocontact shifts; each situation allows the hydration state and relative structure to be inferred.

However, the NMR experiment may be confounded by the lack of solubility in a non-coordinating solvent or there may be fast exchange between the hydrated and non-hydrated structures, not to mention the inherent difficulties of solid-state paramagnetic NMR spectroscopy to provide a reference non-hydrated spectrum.^12^ Here, we highlight a different method, *viz* electron paramagnetic resonance (EPR) spectroscopy, to determine hydration *via* the change in magnetic anisotropy between solid and solution forms of a pair of prototype PARASHIFT reagents.^9,13^ Using cryogenic EPR spectroscopy, we directly probe the ground Kramers doublets of [ErL^1^] (1, where {L^1^}^3-^ = 1,4,7,10-tetraazacyclododecane-1-5-((*tert*-butyl)pyridin-2-yl)methyl-4,7,10-triacetate) and [ErL^2^] (2, where {L^2^}^3-^ = 1,4,7,10-tetraazacyclododecane-1-5-((*tert*-butyl)pyridin-2-yl)methyl-4,7,10-tri(methylphosphinate)),^14^Fig. 1, in their solid and frozen solution phases (subsequently 1_solid_, 1_solution_, 2_solid_ and 2_solution_). We have chosen these Er^III^ complexes because often Er^III^ is not as well sensitised for emission as Tb^III^ or Eu^III^, because Er^III^ is strongly paramagnetic, and because Er^III^ has an odd number of unpaired electrons and as such has a ground state Kramers doublet which is usually EPR active in low symmetry. Using EPR spectroscopy and computational methods, we show unequivocally that 1 binds water in aqueous media while 2 does not. Our results are consistent with the NMR-based determinations of hydration behaviour for the Y^III^ and Yb^III^ analogues of 1 and 2.^13^ The use of EPR spectroscopy to probe hydration effects is equally applicable to any half-integer lanthanide ion (*i.e.* also Ce^III^, Nd^III^, Sm^III^, Gd^III^, Dy^III^ or Yb^III^), however sometimes fast spin-lattice relaxation can broaden spectral lines beyond detection or EPR intensity can be weak owing to small components of EPR-allowed Δ*m*_*S*_ = ±1 states in the ground Kramers doublet; the latter effect is less likely in lower-symmetry complexes.

**Fig. 1:**
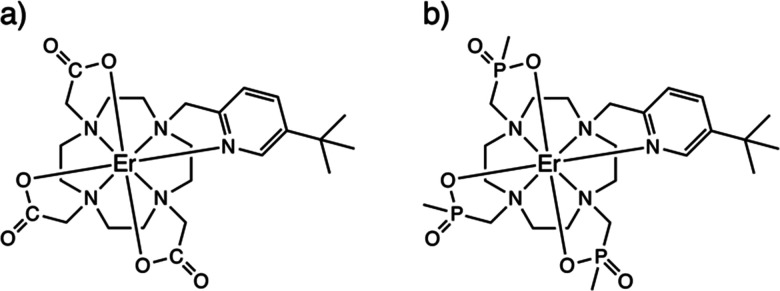
Chemical structures of solid-state (*i.e.* non-hydrated) forms of 1-Λ (a) and 2-Λ (b).

The Er^III^ complexes 1 and 2, as described previously,^13,14^ are both N_4_O_3_N_py_ chelates based on the archetypal DOTA ligand.^15,16^ Functionalisation of one arm to possess a 3-*tert*-butyl-pyridyl group is to provide a strong and distal “reporter” NMR signal, while substitution of the carboxylate groups for phosphinates in L^2^ is designed to provide more steric bulk and prevent solvent coordination. While neither 1_solid_ nor 2_solid_ have been crystallographically characterised, they are presumed to be isostructural with the Yb^III^ analogues [YbL^1^] and [YbL^2^] which crystallise in *P*2_1_/*c* and *P*2_1_, respectively.^13^ The two enantiomers of [YbL^1^] are related by symmetry in the unit cell (the Λ-*λλλλ* and Δ-*δδδδ* forms, where the capital Λ or Δ refers to the helicity of the exocyclic groups, *via* the NCCO(N_py_) torsion angles, and the lower case *λ* or *δ* refers to the NCCN torsions in the 12-N_4_ ring), while the two enantiomers of [YbL^2^] crystallise as independent molecules in the asymmetric unit.^13^

In order to probe directly the ability of L^2^ to protect the coordination sphere of 2 from hydration, in comparison to the less sterically demanding ligand L^1^ in 1, we have performed cryogenic EPR spectroscopic measurements on 1_solid_, 1_solution_, 2_solid_ and 2_solution_. The Q-band EPR spectrum of 1_solid_ at 5 K shows three clear features at *ca.* 0.2, 0.8 and 1.2 T (blue trace, Fig. 2): this is a prototypical rhombic signal of an effective spin *S* = 1/2, where each feature corresponds to a unique *g*-value (*g*_3_, *g*_2_ and *g*_1_, respectively). In the case of Er^III^ in low symmetry, the ground state is a Kramers doublet owing to crystal field splitting of the ^4^I_15/2_ term. We observe no hyperfine coupling to the *I* = 7/2 nuclear spin of ^167^Er (23% natural abundance), likely as it is within the linewidth of the transitions that are broadened by spin-lattice relaxation and strain effects owing to distributions of molecular structure.^17^ Simulation of the spectrum in PHI^18^ using an effective spin *S* = 1/2 model yields effective *g*-values of *g*_1_ = 1.96, *g*_2_ = 3.14 and *g*_3_ = 12.96. Dissolution of 1_solid_ in H_2_O:glycerol 8 : 2, flash freezing at 5 K and repetition of the EPR experiment for 1_solution_ yields a completely different spectrum (green trace, Fig. 2). In this case, the features have collapsed into half the field range of 1_solid_ below 0.8 T. The spectrum is somewhat ambiguous: the second feature at *ca.* 0.4 T could either be one *g* feature alone (and hence the third would be assumed to be out of the field range of the measurement, and thus have *g*_1_ < 1.5), or there could be a shoulder at *ca.* 0.5 T corresponding to the third *g* feature. Hence, we have considered both possibilities: model 1 (purple trace, Fig. 2), assuming all three *g*-values can be observed, can be simulated with an *S* = 1/2 model with *g*-values of *g*_1_ = 4.6, *g*_2_ = 6.59 and *g*_3_ = 9.33; model 2 (yellow trace, Fig. 2), assuming only two *g*-values are observed, gives *g*-values of 
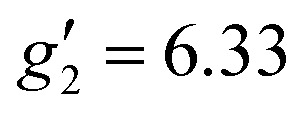
 and 
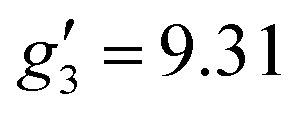
 (where the unobserved *g*-value is fixed to 
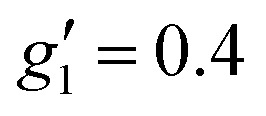
). Model 1 gives three *g*-values for which the sum is quite large (>20), and so it is worthwhile to check that this is physically valid. To do so, we build a crystal field model Hamiltonian of the ^4^I_15/2_ term of Er^III^, parameterised by the values predicted from a complete active space self-consistent field spin-orbit (CASSCF-SO) calculation on a solution-phase structural model of 1_solution_ (see below), and vary the crystal field parameters to fit the effective *g*-values of the ground Kramers doublet to match those obtained in the model 1 simulation. We obtain *g*_1_ = 4.6, *g*_2_ = 6.5 and *g*_3_ = 9.3, suggesting this model is entirely possible (note that the fitted crystal field parameters have no meaning; the model only illustrates that this is a physically possible set of *g*-values for an Er^III^ complex).

**Fig. 2:**
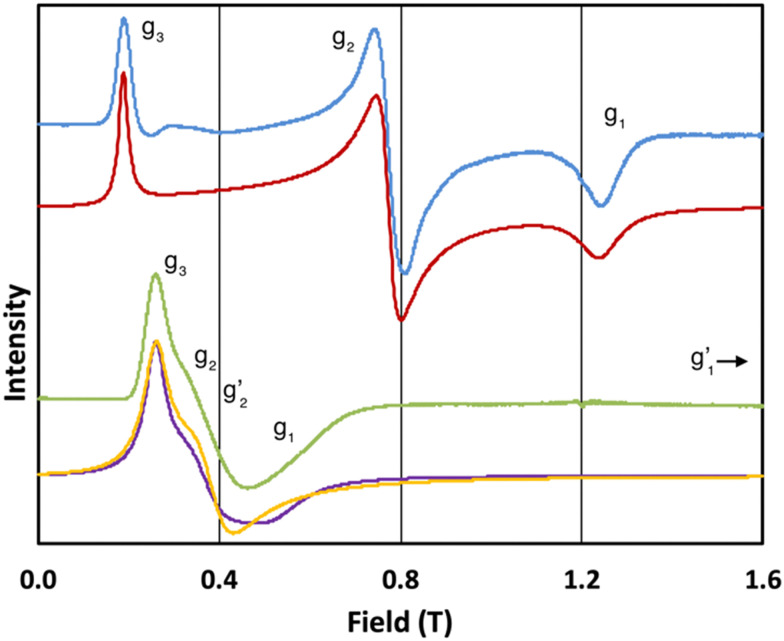
Q-band EPR spectra of 1_solid_ (blue line, 33.993 GHz) and 1_solution_ (green line, 34.092 GHz) at 5 K. Simulations use the parameters in Table 1. For 1_solid_ (red line) anisotropic Lorentzian linewidths (collinear with *g*_1–3_) of 2.9, 2.4 and 4.4 GHz, respectively were included. For 1_solution_ model 1 (purple line) anisotropic Lorentzian linewidths (collinear with *g*_1–3_) of 8.2, 11.5 and 6.5 GHz, respectively, and *g*-strains of 1, 0.8 and 0.4, respectively, were used. For 1_solution_ model 2 (yellow line) an arbitrarily small 
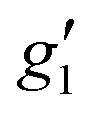
 of 0.4 was used, and anisotropic Lorentzian linewidths (collinear with 
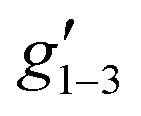
) of 15, 10 and 8.4 GHz, respectively.

The 5 K Q-band spectrum of 2_solid_, shows two resonances at *ca.* 0.2 and 0.8 T (blue trace, Fig. 3), however the resonance at *ca.* 0.8 T does not resemble a *g*_1_ = *g*_2_ feature, indicative that this system has rhombic and not axial symmetry, consistent with the molecular structure and the results for 1_solid_. Simulation gives *g*-values of *g*_2_ = 2.91 and *g*_3_ = 13.67; the third *g*-value is out of the field range of the instrument, and hence *g*_1_ <1.5, and is fixed to 0.4 for the simulation. In contrast to 1, the frozen H_2_O:glycerol sample 2_solution_ shows a very similar spectrum to its solid-state counterpart (green trace, Fig. 3), and simulations give similar *g*-values of *g*_2_ = 3.5 and *g*_3_ = 12.58 (*g*_1_ < 1.5). These spectra immediately demonstrate that the ground state Kramers doublet, and hence the magnetic anisotropy, of 1 changes from the solid-state when in the aqueous phase, while it is practically unchanged for 2. The gross spectral changes for 1, but similar spectra for 2, on dissolution are consistent with hydration in the former case and not the latter.

**Fig. 3:**
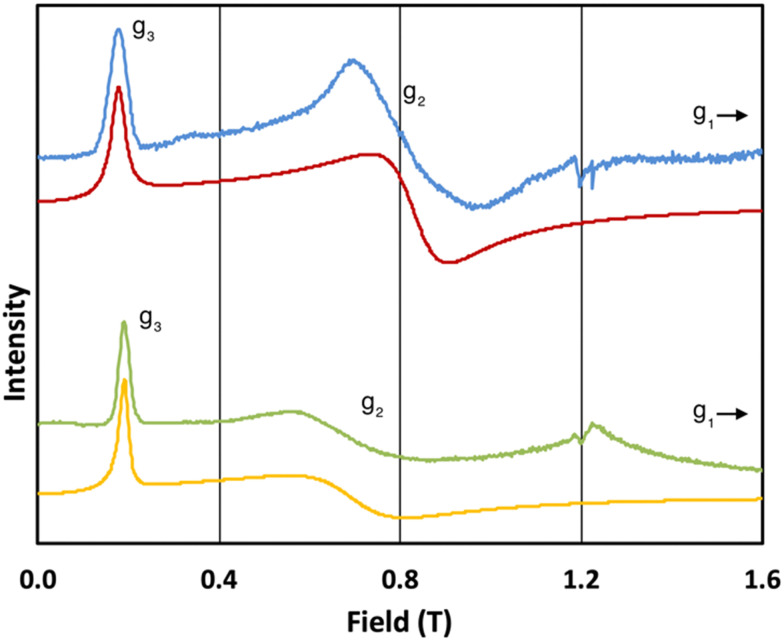
Q-band EPR spectra of 2_solid_ (blue line, 34.180 GHz) and 2_solution_ (green line, 34.016 GHz) at 5 K. Simulations use parameters in Table 1. For 2_solid_ (red line) an arbitrarily small *g*_1_ of 0.4 was used, and anisotropic Lorentzian linewidths (collinear with *g*_1–3_) of 15, 5.3 and 7.2 GHz, respectively. For 2_solution_ (yellow line) an arbitrarily small *g*_1_ of 0.4, and anisotropic Lorentzian linewidths (collinear with *g*_1–3_) of 15, 13 and 4.2 GHz, respectively, were used. (The sharp features at *ca.* 1.2 T are impurities in the cavity, and the broad feature in the experimental data for 2_solution_ centred at 1.2 T is due to the silica capillary.)

To test this hypothesis we sought to affirm the basis of the spectral changes *via* computational methodologies. First, we performed CASSCF-SO calculations in OpenMolcas^19^ using the crystalline geometries of [YbL^1^] and [YbL^2^] with Yb^III^ substituted for Er^III^ (see Methods). These calculations provide a good approximation of the crystal field splitting of the ground ^4^I_15/2_ spin-orbit multiplet for these model complexes (the calculated principal effective *g*-values of all Kramers doublets arising from the ^4^I_15/2_ term are given in Tables S2–S4, ESI†). For 1, the calculated ground Kramers doublet *g*-values are in fair agreement with those found experimentally for 1_solid_ (Table 1), particularly given the structural approximation (noting that Yb^III^ is slightly smaller than Er^III^) and that effective g-values are extremely sensitive to small changes to the orbital composition of the ground doublet and hence structure.^20^ Further discrepancies arise in calculated effective *g*-values due to the approximations inherent in CASSCF-SO (including the lack of dynamic correlation), but are usually <1 for a known structure;^20^ we also note that such minimal active space CASSCF-SO methods tend to over-estimate axiality of the ground Kramers doublet,^21–24^ which we also observe here. For 2, there are two surrogate molecular structures arising from the crystal structure of [YbL^2^] – the Λ-*λλλλ* and Δ-*δδδδ* forms – and calculated ground state *g*-values for both are in fair agreement with the experimental spectrum for 2_solid_ (Table 1). The calculations predict one *g*-value <1, which would be outside the experimental field range, in agreement with experiment.

**Table 1:** EPR *g*-values for 1_solid_, 1_solution_, 2_solid_ and 2_solution_

	Experimental			Calculated			
	*g*_1_	*g*_2_	*g*_3_	*g*_1_	*g*_2_	*g*_3_	Model
1_solid_	1.96	3.14	12.96	1.32	2.39	14.26	[YbL^1^] (ref. 13)
1_solution_	4.6 or < 1.5	6.59 or 6.33	9.33 or 9.31	1.94	5.46	11.60	1A_solution_
				0.93	5.77	11.15	1B_solution_
2_solid_	<1.5	2.91	13.67	0.84	1.59	15.24	[YbL^2^]-Λ-*λλλλ* (ref. 13)
				0.91	1.77	15.19	[YbL^2^]-Δ-*δδδδ* (ref. 13)
2_solution_	<1.5	3.5	12.58	0.29	0.71	16.17	2A_solution_
				0.63	3.11	13.37	2B_solution_

To determine pseudo-solution model structures for 1 and 2, we surrounded the Δ-*δδδδ* forms of each compound in a droplet of 70 water molecules, and then optimised the geometry with the semi-empirical PM6 method in MOPAC.^25,26^ We have done this under two conditions: A) where the whole ensemble is relaxed; and B) where only the solvent is relaxed. This led to two different solvent arrangements, and we found that when the whole ensemble was relaxed for 1 a water molecule coordinated to the Er^III^ ion, but not in the case of 2. Thus, we generated four structures, 1A_solution_, 1B_solution_, 2A_solution_ and 2B_solution_, each of which was further optimised at the density-functional theory (DFT) level (see Methods; Fig. 4 and Fig. S1–S4, ESI†). We find an Er–OH_2_ distance of 2.507 Å for 1A_solution_, which is close to the sum of ionic radii for Er^III^ and O,^27^ and consistent with Er–OH_2_ bond lengths in Er^III^ complexes,^28^ and a longer distance of 2.983 Å for 1B_solution_. These are far shorter than the closest Er–OH_2_ distances in 2A_solution_ and 2B_solution_ which are 3.874 and 4.057 Å, respectively. We then performed CASSCF-SO calculations on the entire ensembles (Tables S5–S8, ESI†). The results for both 1A_solution_ and 1B_solution_ unambiguously show that the close approach of H_2_O in 1 has a significant effect on the magnetic anisotropy of the ground Kramers doublet compared to the solid-state structure, increasing *g*_*2*_ by 3.2 and decreasing *g*_3_ by 2.9 on average, towards the experimental values. We find that 1A_solution_ seems to support the model 1 simulations of 1_solution_, where *g*_1_ increases from the crystal structure, while 1B_solution_ seems to support the model 2 simulations of 1_solution_ where *g*_1_ is <1.5. From these results we cannot definitively conclude which simulation of the experimental spectrum is more accurate, however it is clear that the close approach or coordination of a water molecule to Er^III^ in 1_solution_ leads to a drastic change in magnetic anisotropy. On the other hand, the not-so-close approaches of H_2_O to Er^III^ in 2 lead to smaller changes compared to the solid-state structures, where we see both *g*_2_ and *g*_3_ changing by ±1.3 on average. Here, the best agreement with experiment is for 2B_solution_, where the nearest water molecule is further away from the Er^III^ ion than in 2A_solution_.

**Fig. 4:**
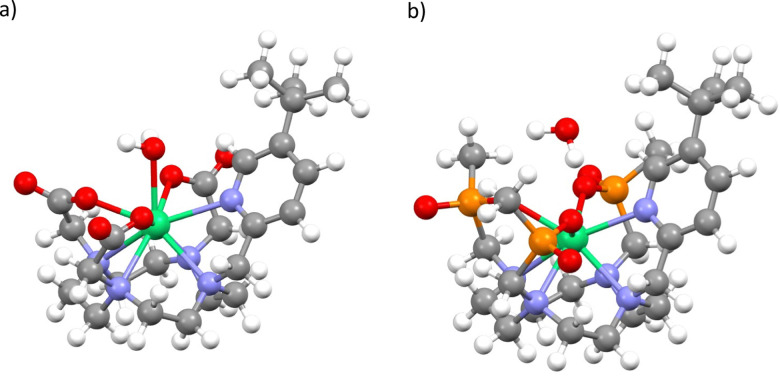
Optimised structure of 1A_solution_ (a) and 2A_solution_ (b). Note: most explicit water not shown, see Fig. S1 and S3 (ESI†).

Thus, we have confirmed using EPR spectroscopy that the trimethylphosphinate ligand L^2^ in compound 2 effectively protects the coordination sphere of the bound erbium ion from hydration, whereas the triacetate ligand L^1^ in compound 1 is not sufficiently bulky to prevent water coordination, and this compound is certainly hydrated in aqueous solution. This work highlights the utility of EPR as a complimentary technique to NMR and luminescence methods for determination of hydration and solution structure.

We thank EPSRC (EP/N007034/1), The Royal Society (URF191320) and the ERC (ERC-2019-STG-851504) for funding. We thank the Computational Shared Facility at The University of Manchester for access to the CSF3. We thank the EPSRC EPR National Facility for access to EPR spectroscopy (EP/W014521/1 and EP/V035231/1). The data that support the findings of this study are openly available in FigShare at https://doi.org/10.48420/21740702.

EJLM, DP and NFC conceived the project. KM synthesised the compounds. NFC measured EPR spectra. MJG, MV, YLW, CW and NFC performed calculations. NFC wrote the manuscript with input from all authors.

## Conflicts of interest

There are no conflicts to declare.

## Supplementary Material

CC-059-D3CC00601H-s001
