# Supplementary material for: Determination of molecular hydration in solution via changes in magnetic anisotropy
Source: Chem Commun (Camb). 2023 Mar 20;59(30):4531–4. doi: 10.1039/d3cc00601h (PMC10088811; doi:10.1039/d3cc00601h)
Supplement: CC-059-D3CC00601H-s001 [file CC-059-D3CC00601H-s001.pdf]

Electronic Supplementary Material (ESI) for ChemComm.
This journal is © The Royal Society of Chemistry 2023

Supplementary information for Determination of molecular hydration in solution via changes in magnetic anisotropy

Marcus J. Giansiracusa,[1] Michele Vonci,[1] Yasmin L. Whyatt,[1] Carys Williams,[1] Kevin Mason,[2] David Parker,[2,*] Eric J. L. McInnes[1,*] and Nicholas F. Chilton[1,*]

[1] Department of Chemistry, The University of Manchester, Oxford Road, Manchester, M13 9PL, UK
[2] Department of Chemistry, Durham University, South Road, Durham, DH1 3LE, UK

Experimental methods

Proton and ^{31}P NMR spectra were recorded in commercially-available deuterated solvents on a Varian Mercury-400 (^{1}H at 399.960 MHz and ^{31}P at 161.943 MHz), or a Varian VNMRA-700 (^{1}H at 699.731 MHz, ^{31}P at 283.256 MHz) spectrometer. All chemical shifts are given in ppm and coupling constants are reported in Hz. Where required, the operating temperature of the spectrometer was measured with the aid of an internal calibration solution of ethylene glycol.

Low resolution electrospray mass spectra were obtained on a TQD mass spectrometer, operating in positive or negative ion mode, equipped with an Acquity UPLC, an electrospray ion source and an Acquity photodiode array detector (Waters Ltd, UK). High resolution electrospray mass spectra were recorded on a LCT Premier XE mass spectrometer or a QTOF Premier mass spectrometer, both equipped with an Acquity UPLC, a lock-mass electrospray ion source and an Acquity photodiode array detector (Waters Ltd, UK).

Reverse phase HPLC was performed at 295 K using a Shimadzu system consisting of a Degassing Unit (DGU-20A5R), a Prominence Preparative Liquid Chromotograph (LC-20AP), a Prominence UV/Vis Detector (SPD-20A) and a Communications Bus Module (CBM-20A). An XBridge C18 19 x 100 mm, i.d. 5 μM column was used to purify the erbium(III) complexes on a preparative scale (flow rate 17 mL/min) and an XBridge C18 column, 4.6 x 100 mm, i.d. 5 μM (flow rate 2 mL/min) was used for analytical scale purifications. A gradient elution with a solvent system composed of H2O + 0.1% HCOOH/ MeOH + 0.1% HCOOH was performed for a total run time of 17 min. The separation details are reported below (Table S1). Fraction collection was performed manually.

Table S1. Preparative HPLC Procedure (flow rate = 17 mL/min; Solvent A = H_2O + 0.1% formic acid; Solvent B = MeOH + 0.1% formic acid).

Time (min)	Solvent A (%)	Solvent B(%)
0	90	10
3	90	10
13	0	100
16	0	100
17	90	10

EPR spectroscopy was performed on powder samples of $\mathbf{1}_{\mathrm{solid}}$ and $\mathbf{2}_{\mathrm{solid}}$ in quartz EPR tubes, and frozen solutions (H_2O:glycerol 8:2) of $\mathbf{1}_{\mathrm{solution}}$ and $\mathbf{2}_{\mathrm{solution}}$ in silica capillaries, at Q-band frequency (ca. 34 GHz) with a Bruker EMX spectrometer using liquid helium cooling.

Semi-empirical optimisations were performed on hydrated cluster models of **1** and **2** using MOPAC with the PM6 method,[3,4] where either the Er^{III} complex was frozen or not. Subsequent DFT optimisations of these hydrated cluster models, where Er was replaced with Y, were performed using Gaussian 09d,[5] the M06-2X functional,[6] the cc-pVDZ basis set[7] for all non-metal atoms, the Stuttgart RSC 1997 ECP[8] for Y, the SMD continuum solvent model,[9] and GD3[10] dispersion corrections. Due to the many degrees of freedom and shallow potential energy surface, it was not possible to fully converge the DFT optimisations; residual forces were on the order of 2.0×10^{-2} eV $Å^{-3}$ compared to the standard convergence threshold of 1.5×10^{-2} eV $Å^{-3}$. CASSCF-SO calculations were performed using OpenMolcas[11] where the active space comprised eleven 4f electrons in seven 4f orbitals of Er^{III} for 35 quartet and 112 doublet roots. Basis sets were taken from the ANO-RCC library where Er atom had VTZP quality, coordinated O and N atoms had VDZP quality, remaining atoms had VDZ quality and surrounding waters had MB quality.[12–14] The two electron integrals were decomposed using the Cholesky method with a threshold of 10^{-8} to save computational resources.

Synthesis of 1 and 2

The syntheses of *tert*-Butyl-2,2',2''-(10-((5-tert-butylpyridin-2-yl)methyl)-1,4,7,10-tetraazacyclododecane-1,4,7-triyl)triacetate (L^1) and ethyl ({4-[(5-*tert*-butylpyridin-2-yl)methyl]-7,10-di{[ethoxy(methyl)phosphoryl]methyl}-1,4,7,10-tetraazacyclododecan-1-yl}methyl)(methyl)phosphinate (L^2) are given in references 1 and 2.

*[ErL^1] (**1**):*

tert-Butyl-2,2',2''-(10-((5-tert-butylpyridin-2-yl)methyl)-1,4,7,10-tetraazacyclododecane-1,4,7-triyl)triacetate (100 mg, 0.15 mmol) was dissolved in CH_2Cl_2 (1 mL) and to this was added TFA (1 mL). The resulting mixture was stirred at room temperature for 18 h. Excess CH_2Cl_2 and TFA were removed under reduced pressure and the resulting yellow residue was repeatedly washed with CH_2Cl_2 (3 x 1 mL). Deprotection was monitored by ESI-LRMS (+) (m/z 494.2 $[M+H]^+$; ESI-HRMS (+) calc. for $[C_{24}H_{40}N_5O_6]^+$ 494.2979, found 494.2963) and complete loss of the t-butyl groups was confirmed by ^1H NMR spectroscopy. The residue was dissolved in H_2O (2 mL) and the pH adjusted to 5.5. To this solution was added $ErCl_3.6H_2O$ (63mg, 0.17 mmol), and the resulting mixture was stirred at 70 ° C for 18 h, before the solvent was removed under reduced pressure. The resulting yellow solid was purified by reverse-phase HPLC (10-100% MeOH in H_2O over 10 min; t_R = 8.9 min) to yield a white solid (29 mg, 29%). ^1H NMR (400 MHz, D_2O) δ 7.0 ($C(CH_3)_3$); ESI-LRMS (+) m/z 657.1 $[MH]^+$; ESI-HRMS (+) calc. for$[C_{24}H_{37}N_5O_6{}^{166}Er]^+$ 657.2047, found 657.2045.

*[ErL^2] (**2**):*

Ethyl ({4-[(5-*tert*-butylpyridin-2-yl)methyl]-7,10-di{[ethoxy(methyl)phosphoryl]methyl}-1,4,7,10-tetraazacyclododecan-1-yl}methyl)(methyl)phosphinate (45 mg, 0.065 mmol) was dissolved in aqueous HCl (6M, 2 mL) and stirred at 70 ° C for 18 h. Complete ester hydrolysis was confirmed by ^{31}P NMR spectroscopy ((162 MHz, D_2O) δ 37.0, 34.4, 30.6), before the solvent was removed under reduced pressure. The resulting yellow oil was dissolved in H_2O (2 mL) and the pH of the solution adjusted to 5.5 using aqueous sodium hydroxide solution. $ErCl_3.6H_2O$ (37 mg, 0.098 mmol), was added and the solution was stirred at 70 ° C for 18 h before solvent was removed under reduced pressure. The resulting pale yellow solid was purified by reverse-phase HPLC (10-100% MeOH in H_2O over 10 min; t_R = 8.6 min) to yield a colourless solid (16 mg, 32%). ^1H NMR (400MHz, D_2O) δ 38.3 ($C(CH_3)_3$ major), 40.1 ($C(CH_3)_3$ minor); ESI-LRMS (+) m/z 758.9$[M+H]^+$; ESI-HRMS (+) calc. for $[C_{24}H_{46}N_5O_6P_3{}^{164}Er]$+ 757.1953, found 757.1962.

Table S2. Electronic states for non-hydrated crystalline **1**-Δ-δδδδ (using X-Ray structure of [YbL[1]]) calculated by CASSCF-SO.

**Energy (cm^{-1})**	g_1	g_2	g_3
0.0	1.32	2.39	14.26
60.8	1.00	1.91	15.22
106.4	2.52	4.74	8.41
157.4	0.17	2.36	12.03
295.8	1.09	1.17	14.36
379.1	2.78	5.91	7.81
425.8	2.37	4.82	8.94
464.5	1.46	2.56	14.31

Table S3. Electronic states for non-hydrated crystalline **2**-Δ-δδδδ (using X-Ray structure of [YbL[2]]) calculated by CASSCF-SO.

**Energy (cm^{-1})**	g_1	g_2	g_3
0.0	0.91	1.77	15.19
45.6	2.09	3.05	13.92
90.8	1.24	4.06	8.49
120.6	0.52	3.29	12.12
222.3	0.04	0.90	15.22
320.6	2.08	4.37	9.95
357.4	1.56	4.48	10.78
380.4	1.21	1.87	15.20

Table S4. Electronic states for non-hydrated crystalline **2**-Λ-λλλλ (using X-Ray structure of [YbL[2]]) calculated by CASSCF-SO.

**Energy (cm^{-1})**	g_1	g_2	g_3
0.0	0.84	1.59	15.24
44.0	1.91	2.64	14.37
88.2	1.89	4.81	8.56
122.2	0.66	2.73	12.47
221.4	0.19	0.93	15.23
314.4	0.77	1.92	13.01
365.5	0.23	2.36	13.76
387.2	0.92	1.55	15.68

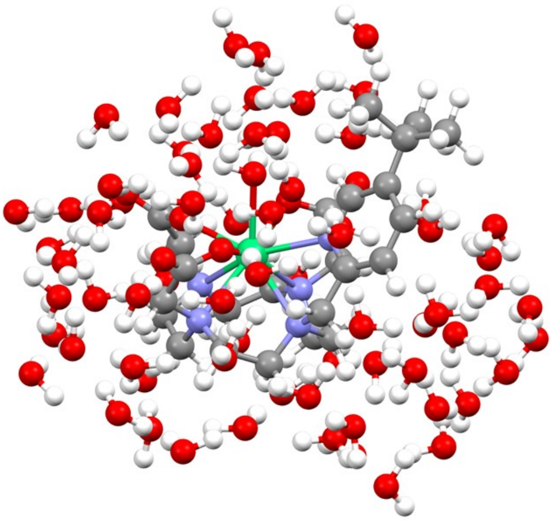

Figure S1. Optimised structure of $\mathbf{1A}_{\mathbf{solution}}$.

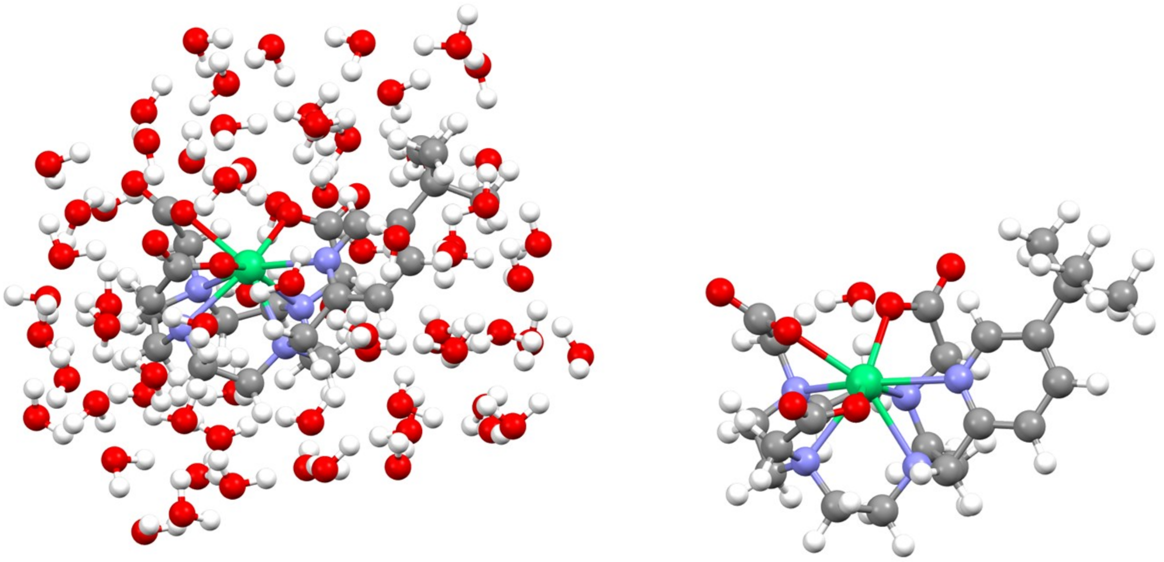

Figure S2. Optimised structure of $\mathbf{1B}_{\mathbf{solution}}$. Left: full structure; right: coordination complex only.

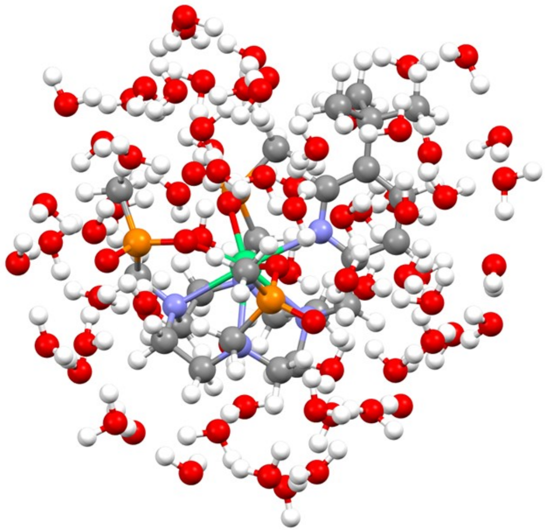

Figure S3. Optimised structure of $\mathbf{2A}_{\mathbf{solution}}$.

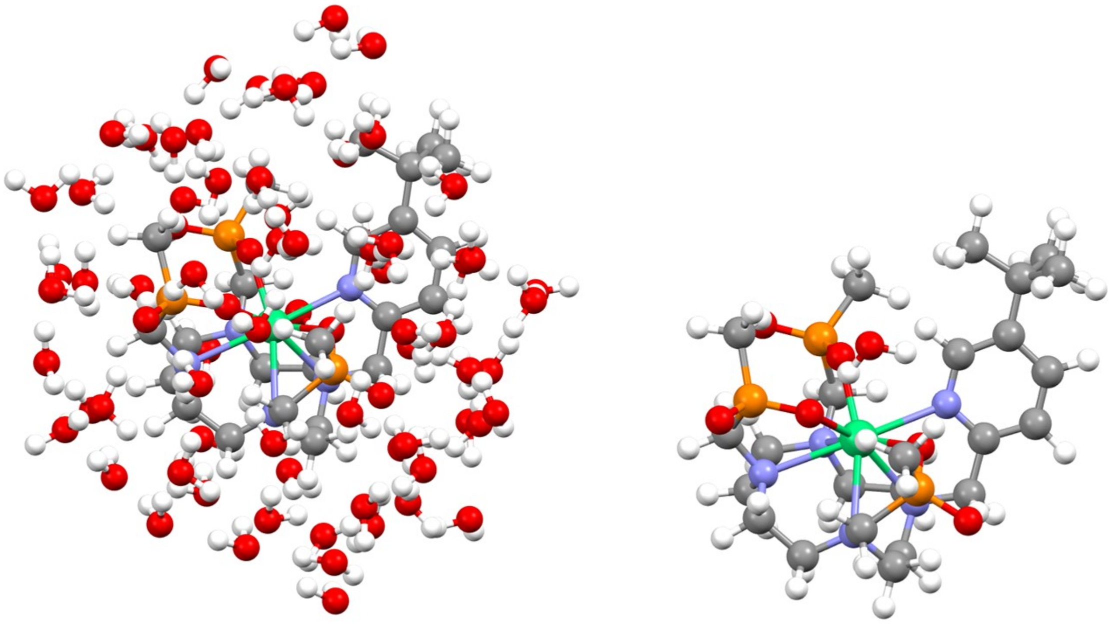

Figure S4. Optimised structure of $\mathbf{2B}_{\mathrm{solution}}$. Left: full structure; right: coordination complex only.

Table S5. Electronic states for DFT-optimised $\mathbf{1A}_{\mathrm{solution}}$ calculated by CASSCF-SO.

Energy (cm^{-1})	g_1	g_2	g_3
0.0	1.94	5.46	11.60
43.9	2.70	3.48	5.52
74.2	0.02	4.37	9.14
110.2	1.87	3.07	9.63
163.9	0.11	2.98	10.86
223.0	0.19	3.56	11.39
259.2	2.31	3.50	10.84
305.0	0.90	1.32	14.31

Table S6. Electronic states for DFT-optimised $\mathbf{1B}_{\mathrm{solution}}$ calculated by CASSCF-SO.

Energy (cm^{-1})	g_1	g_2	g_3
0.0	0.93	5.77	11.15
26.9	1.23	3.62	12.55
66.5	3.64	5.48	9.51
109.6	0.15	1.18	11.83
211.0	1.40	2.84	10.36
246.3	0.05	6.17	9.99
275.1	2.90	3.61	10.50
335.5	0.60	0.79	15.63

Table S7. Electronic states for DFT-optimised $\mathbf{2A}_{\mathbf{solution}}$ calculated by CASSCF-SO.

Energy (cm^{-1})	g_1	g_2	g_3
0.0	0.29	0.71	16.17
28.8	1.02	1.36	16.13
88.0	3.32	6.28	7.40
134.7	1.09	3.11	11.89
247.2	1.41	2.25	13.61
283.3	0.15	3.78	9.64
321.8	1.30	3.29	12.01
347.6	1.35	1.73	14.56

Table S8. Electronic states for DFT-optimised $\mathbf{2B}_{\mathbf{solution}}$ calculated by CASSCF-SO.

Energy (cm^{-1})	g_1	g_2	g_3
0.0	0.63	3.11	13.37
18.6	1.43	1.81	15.62
76.0	2.24	4.57	7.43
106.6	1.48	4.47	11.76
236.7	1.41	2.33	13.24
276.2	3.02	4.57	7.96
310.6	1.88	4.92	10.99
353.9	0.51	0.53	16.85

References

1 A. M. Funk, K.-L. N. A. Finney, P. Harvey, A. M. Kenwright, E. R. Neil, N. J. Rogers, P. Kanthi Senanayake and D. Parker, *Chem Sci*, 2015, **6**, 1655–1662.

2 K. Mason, N. J. Rogers, E. A. Suturina, I. Kuprov, J. A. Aguilar, A. S. Batsanov, D. S. Yufit and D. Parker, *Inorg. Chem.*, 2017, **56**, 4028–4038.

3 J. J. P. Stewart, .

4 J. J. P. Stewart, *J. Mol. Model.*, 2007, **13**, 1173–1213.

5 M. J. Frisch, G. W. Trucks, H. B. Schlegel, G. E. Scuseria, M. A. Robb, J. R. Cheeseman, G. Scalmani, V. Barone, B. Mennucci, G. A. Petersson, H. Nakatsuji, M. Caricato, X. Li, H. P. Hratchian, A. F. Izmaylov, J. Bloino, G. Zheng, J. L. Sonnenberg, M. Hada, M. Ehara, K. Toyota, R. Fukuda, J. Hasegawa, M. Ishida, T. Nakajima, Y. Honda, O. Kitao, H. Nakai, T. Vreven, J. A. Montgomery Jr., J. E. Peralta, F. Ogliaro, M. Bearpark, J. J. Heyd, E. Brothers, K. N. Kudin, V. N. Staroverov, T. Keith, R. Kobayashi, J. Normand, K. Raghavachari, A. Rendell, J. C. Burant, S. S. Iyengar, J. Tomasi, M. Cossi, N. Rega, J. M. Millam, M. Klene, J. E. Knox, J. B. Cross, V. Bakken, C. Adamo, J. Jaramillo, R. Gomperts, R. E. Stratmann, O. Yazyev, A. J. Austin, R. Cammi, C. Pomelli, J. W. Ochterski, R. L. Martin, K. Morokuma, V. G. Zakrzewski, G. A. Voth, P. Salvador, J. J. Dannenberg, S. Dapprich, A. D. Daniels, Ö. Farkas, J. B. Foresman, J. V. Ortiz, J. Cioslowski and D. J. Fox, .

6 Y. Zhao and D. G. Truhlar, *Theor. Chem. Acc.*, 2008, **120**, 215–241.

7 T. H. Dunning, *J. Chem. Phys.*, 1989, **90**, 1007–1023.

8 M. Dolg, H. Stoll, H. Preuss and R. M. Pitzer, *J. Phys. Chem.*, 1993, **97**, 5852–5859.

9 A. V. Marenich, C. J. Cramer and D. G. Truhlar, *J. Phys. Chem. B*, 2009, **113**, 6378–6396.
10 S. Grimme, J. Antony, S. Ehrlich and H. Krieg, *J. Chem. Phys.*, 2010, **132**, 154104.
11 I. Fdez. Galván, M. Vacher, A. Alavi, C. Angeli, F. Aquilante, J. Autschbach, J. J. Bao, S. I. Bokarev, N. A. Bogdanov, R. K. Carlson, L. F. Chibotaru, J. Creutzberg, N. Dattani, M. G. Delcey, S. S. Dong, A. Dreuw, L. Freitag, L. M. Frutos, L. Gagliardi, F. Gendron, A. Giussani, L. González, G. Grell, M. Guo, C. E. Hoyer, M. Johansson, S. Keller, S. Knecht, G. Kovačević, E. Källman, G. Li Manni, M. Lundberg, Y. Ma, S. Mai, J. P. Malhado, P. Å. Malmqvist, P. Marquetand, S. A. Mewes, J. Norell, M. Olivucci, M. Oppel, Q. M. Phung, K. Pierloot, F. Plasser, M. Reiher, A. M. Sand, I. Schapiro, P. Sharma, C. J. Stein, L. K. Sørensen, D. G. Truhlar, M. Ugandi, L. Ungur, A. Valentini, S. Vancoillie, V. Veryazov, O. Weser, T. A. Wesołowski, P.-O. Widmark, S. Wouters, A. Zech, J. P. Zobel and R. Lindh, *J. Chem. Theory Comput.*, 2019, **15**, 5925–5964.
12 B. O. Roos, R. Lindh, P.-Å. Malmqvist, V. Veryazov and P.-O. Widmark, *Chem. Phys. Lett.*, 2005, **409**, 295–299.
13 B. O. Roos, R. Lindh, P.-Å. Malmqvist, V. Veryazov and P.-O. Widmark, *J. Phys. Chem. A*, 2005, **109**, 6575–6579.
14 B. O. Roos, R. Lindh, P.-Å. Malmqvist, V. Veryazov and P.-O. Widmark, *J. Phys. Chem. A*, 2004, **108**, 2851–2858.
